# Validation Study of ASA Physical Status Prediction Model Developed Through Holistic Integration of Lung Images, Electrocardiogram Data and Clinical Data

**DOI:** 10.3390/jcm15207965

**Published:** 2026-10-15

**Authors:** Kadir Yildirim, Serpil Bayindir, Mucahit Karaduman, Muhammed Yildirim

**Affiliations:** 1 Department of Urology, Elazig Fethi Sekin City Hospital, University of Health Sciences, 23280 Elazig, Türkiye; 2 Clinics of Anesthesiology and Reanimation, Elazig Fethi Sekin City Hospital, 23280 Elazig, Türkiye; serpil.bayindir@saglik.gov.tr; 3 Department of Software Engineering, Malatya Turgut Ozal University, 44210 Malatya, Türkiye; mucahit.karaduman@ozal.edu.tr; 4 Department of Artificial Intelligence and Data Engineering, Firat University, 23119 Elazig, Türkiye; muhammedyildirim@firat.edu.tr

**Keywords:** objective ASA scoring, posteroanterior chest X-ray, ECG, CNN, clinical decision support

## Abstract

**Background/Objectives**: Objective assessment of patients’ physical health status before surgical interventions is crucial in predicting surgical risks and planning patient management. The American Society of Anesthesiologists Physical Status Classification (ASA-PS) is one of the most widely used scales in this context. It plays a vital role in clinical decision-making processes. However, ASA scoring can vary among clinicians due to its reliance on subjective assessments, making it difficult to standardize. **Methods**: In this study, a deep learning-based model was developed to classify ASA from patient data automatically. Three different data types were used in this study: lung images, Electrocardiogram (ECG) data, and clinical data. For the proposed ASA prediction model, features were extracted from each data type. Features were extracted from ECG and Computed Tomography (CT) images using five different Convolutional Neural Network (CNN) architectures, while statistical features were derived from the clinical data. The features obtained from the CNN architecture with the highest classification performance were combined with the statistical features. These combined features were then processed using seven different classifiers. **Results**: The developed model was evaluated with performance metrics such as accuracy, precision, F1 score, and Receiver Operating Characteristic (ROC) curve. As a result, a high accuracy value of 97.30% was obtained. **Conclusions**: This study aims to make preoperative risk management for ASA classification more objective and consistent, and the results obtained support this aim.

## 1. Introduction

The American Society of Anesthesiologists Physical Status (ASA-PS) classification system, a guide for anesthesiologists, was first published in 1941 by Dr. Meyer Saklad et al. as a method for collecting and categorizing anesthesia statistical data. It was proposed by Dripps et al. in 1961 and accepted by the ASA in 1962 [1]. In this version, classes 1 to 4 were retained, and instead of separate classes used for emergencies, the practice of adding “E” (Emergency) to any ASA class was introduced. The most current and official version of the ASA-PS classification, which has been used for approximately 65 years, is found in the document titled “Statement on ASA Physical Status Classification System” published by the ASA on its official website. The ASA House of Delegates approved this document and it is regularly reviewed and updated [2,3].

Accurately assessing the anesthesia risk of patients in the preoperative period and making the most appropriate preoperative preparation according to the results are of critical importance for safe surgical processes. In this respect, the ASA-PS classification serves as a fundamental tool that guides the planning and management of operations by categorizing patients’ general physical condition into understandable and straightforward categories. However, since the ASA-PS classification requires multi-dimensional thinking by combining multiple parameters and sometimes involves a subjective decision process, differences may occur between evaluators, and it may not be easy to reach a consensus [4]. This situation may create potential difficulties in correctly estimating the patient’s risk and thus determining the most appropriate anesthesia management.

Today, Artificial Intelligence (AI) and its sub-branches, machine learning (ML), fuzzy logic, and neural networks, offer significant capabilities in analyzing complex data and making predictions. Integrating machine learning algorithms into preoperative workflows has enabled the detection of previously unnoticed complex relationships by creating virtual networks [5]. ML systems that can analyze complex medical data by combining biophysics and computer science have also triggered significant technological advances in the field of medicine [6]. These technologies train models using large and heterogeneous datasets, such as clinical patient data, to obtain the desired outputs. In the field of anesthesia, this potential of AI offers an opportunity for more accurate, rapid, and optimal determination of ASA-PS classification [7].

In this study, we aimed to develop an ML algorithm that predicts ASA-PS scores by combining biochemical data, demographic features, and imaging results, including electrocardiography (ECG) and posteroanterior (PA) chest X-rays. Most studies on ASA classification have been limited to numerical data (demographic information, laboratory results, etc.). The integration of data, such as lung images and ECG signals, has often been overlooked. In this study, the model’s performance is enhanced by combining complementary information from different data types using the multimodal data approach. In this context, automatic ASA classification by evaluating image, signal, and clinical data together not only increases the classification accuracy but also increases the reliability of clinical decision support systems. In this study, a model that performs automatic ASA-PS classification by integrating three different data types in a deep learning-based architecture has been developed and validated. In addition, this study aims to reduce subjectivity in anesthesia risk assessment by offering a different perspective on the approach and obtaining more objective and consistent results. Therefore, the aim is to improve preoperative patient management and contribute to increasing surgical safety.

## 2. Methodology

**Figure 1:**
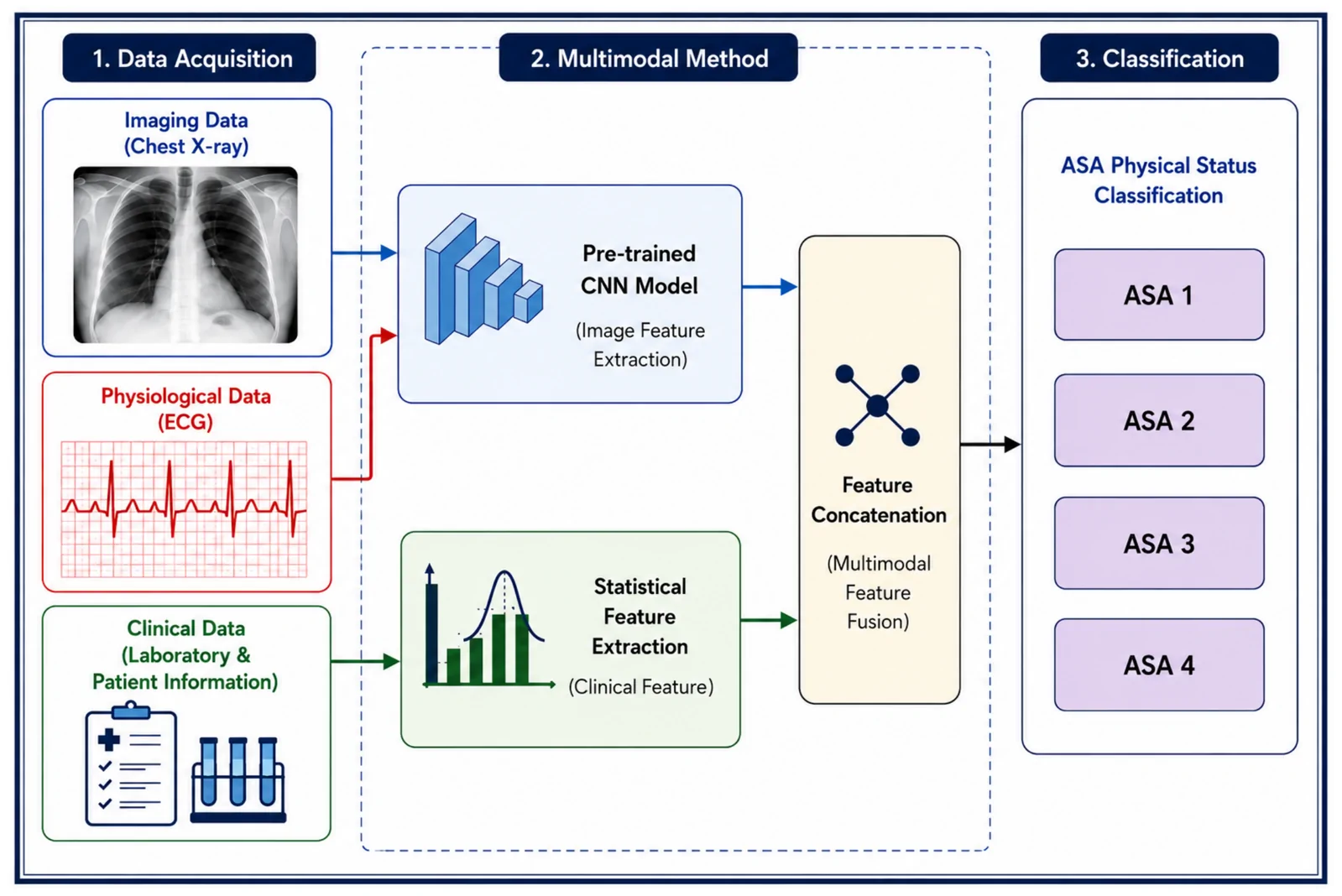
Flow chart of proposed model.

In this section, the models and methods used in the study are discussed. Additionally, the dataset used for the study is provided. A rough flow chart of the study is presented in Figure 1.

### 2.1. Study Design and Settings

This was a retrospective, single-center clinical study designed to evaluate the ASA-PS classification in patients undergoing elective surgery. Approval for the study was obtained from the Elazig Fethi Sekin City Hospital Non-Interventional Clinical Research Ethics Committee (Decision No: 2025/11-34, Date: 12 June 2025). All procedures adhered strictly to the principles outlined in the Declaration of Helsinki. In this retrospective study, ASA-PS classifications were derived from existing ASA-PS records in patients’ clinical files during routine preoperative evaluations. Patients were not reclassified according to ASA-PS classifications within the scope of the study. During the data transfer to the dataset, an anesthesiologist reviewed the existing ASA-PS records and related clinical information to prevent possible recording or coding errors.

### 2.2. Participants

Patients who applied to the anesthesia clinic for elective surgery were included in this study. ASA-PS scores range from 1 to 6 and are determined by anesthesiologists during preanesthetic evaluation. In our hospital, ASA 5 and ASA 6 patients were excluded from the study to prevent unbalanced data distribution, as they comprised a small number of surgeries and were predominantly in the emergency surgery group. Other exclusion criteria included patients who would undergo emergency surgery, those under the age of 18, and pregnant patients (since a PA chest X-ray would be used in the evaluation). The inclusion criteria were patients over the age of 18 who applied to the anesthesia clinic for elective surgery and whose data were not missing.

### 2.3. Protocol and Outcome Measures

**Figure 2:**
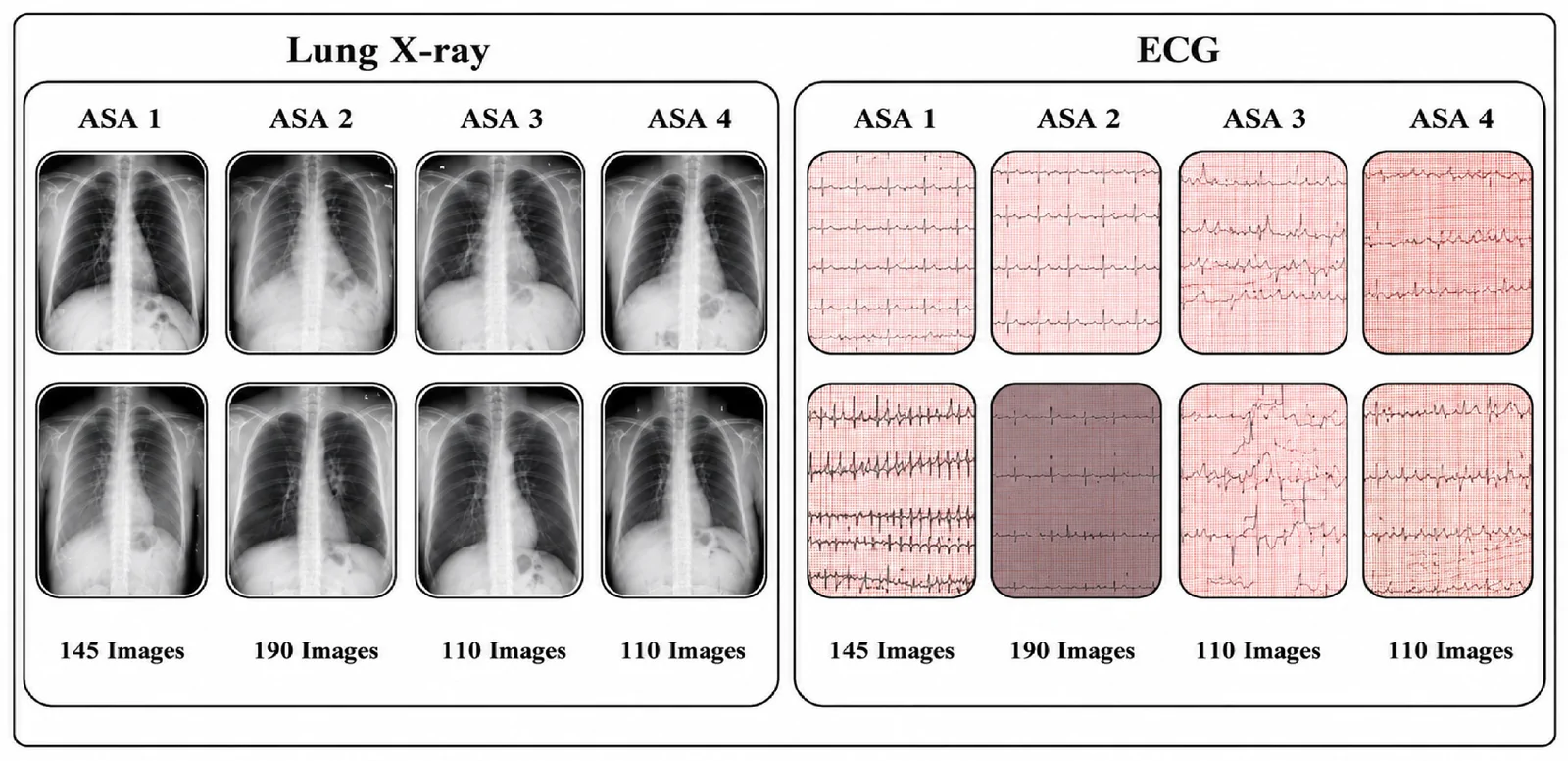
Examples of PA Lung X-rays and ECG signals.

Patients who applied for preanesthetic evaluation and met the inclusion criteria were risk-assessed by the same anesthesiologist using the current ASA-PS classification. In the evaluation, anamnesis, biochemical data, a PA chest X-ray, and an ECG were interpreted and utilized. PA chest X-rays were obtained from the Hospital Information Management System after obtaining the necessary approvals from the local ethics committee. During this process, the Personal Data Protection Law (KVKK) and relevant medical data privacy regulations in Türkiye were strictly adhered to. ECG images were physically retrieved from the hospital’s archive files and transferred to a digital environment. During this procurement process, all legal and ethical standards regarding patient privacy and data security were fully complied with. The primary outcome measure is how accurately and reliably the machine learning model we developed predicts the ASA-PS classification. Accuracy, sensitivity, specificity, precision, F1 score, and AUC/ROC curves were used to measure the performance of the models used in the study. Examples of PA Lung X-rays and ECG signals used in the study are presented in Figure 2.

In our study, biochemical data (glucose, urea, creatinine, AST, ALT, bilirubin, Na, K, Cl, TFT, Hb, Htc, Plt, INR, PTZ, APTT, HBsAg, HCV, AntiHIV) together with clinical and demographic data (smoking, alcohol use, Body Mass Index (BMI), presence of systemic disease, presence of a pacemaker, ejection fraction (EF), need for dialysis, Myocardial Infarction (MI), cerebrovascular accident (CVA), Transient Ischemic Attack (TIA), hypertension (HT), Coronary Artery Disease (CAD), valve disease, Ischemic Heart Disease (IHD), shock, sepsis, Disseminated Intravascular Coagulation (DIC) and Acute Respiratory Distress Syndrome (ARDS)) were used for ASA-PS estimation.

Statistical features, including mean, variance, standard deviation, skewness, and kurtosis, were obtained from the clinical and laboratory data of each patient. These five statistical features were combined with depth features extracted from chest X-ray and ECG images using pre-trained CNN models to create a multimodal feature vector. In this coding process, the current ASA-PS classification, as determined by the American Society of Anesthesiologists (ASA) and included in the most recent “Statement on ASA Physical Status Classification System” document, served as the basis.

The ASA-PS status of each patient was processed into the dataset by assigning numerical values according to the following criteria:

ASA-PS 1: Healthy individuals are included in this category. Patients included in this group are normal healthy individuals who do not smoke, consume little or no alcohol, and do not have a known systemic disease.

ASA-PS 2: Patients with mild systemic conditions or diseases are included in this category. These individuals have mild conditions or diseases that do not cause significant functional limitations. Examples include patients with active smoking, social alcohol consumption, pregnancy status, obesity (BMI 30–40), well-regulated DM or HT, or mild lung disease.

ASA-PS 3: Patients with severe systemic disease that is not a permanent threat to life are included in this category. This group encompasses one or more moderate to severe diseases that result in significant functional limitations. Examples include poorly controlled diabetes or hypertension, COPD, morbid obesity (BMI ≥ 40), active hepatitis, alcohol dependence or abuse, presence of an implanted pacemaker, moderate reduction in ejection fraction, end-stage renal disease on regular dialysis, MI, CVA, TIA or CAD/stent placement more than 3 months ago.

ASA-PS 4: Patients with severe systemic disease that is a constant threat to life are included in this category.

This standardized coding provided a fundamental data structure for accurately training the machine learning model and objectively predicting the ASA-PS classification.

### 2.4. Proposed Model

**Figure 3:**
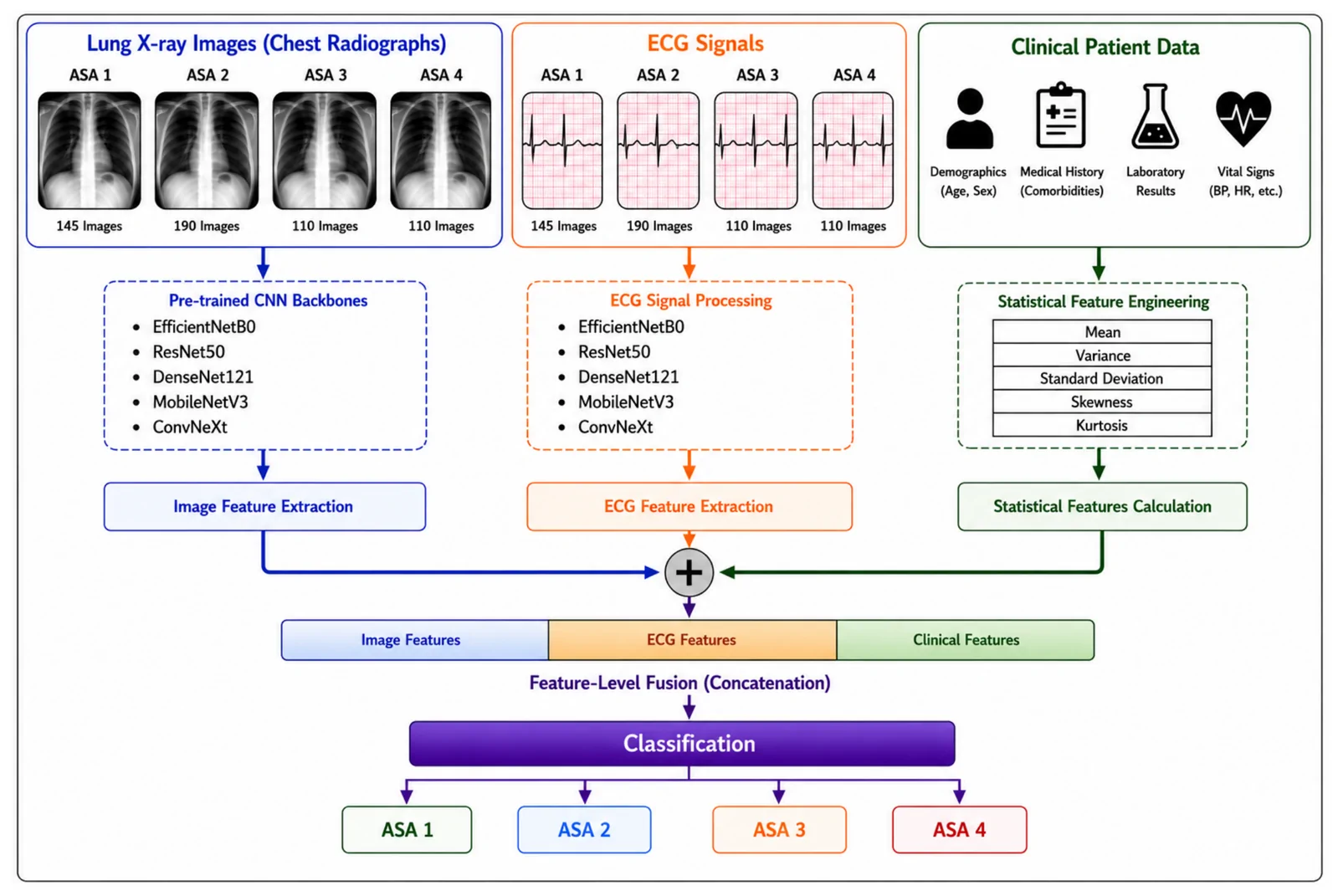
General structure of the proposed system for ASA prediction.

In this study, a multimodal method is proposed using three different datasets taken from the same patients. The data taken from the patients consist of lung images, ECG data, and clinical data. The general structure of the proposed system for ASA estimation is given in Figure 3.

In Figure 3, lung images are passed through pre-trained models according to patient number and class and then features are extracted. The exact process is performed for ECG image data. Finally, the patient’s clinical data are taken, and statistical features such as mean, variance, standard deviation, skewness, and kurtosis are extracted. After the features are extracted according to the patient number, a feature vector is extracted for each patient by merging. The extracted feature vectors are given to the classification algorithms, and the classification process is performed. After this process, accuracy, precision, recall, and F1-scores are examined for performance analysis.

Five different deep learning models are employed to compare the successes of these models in the feature extraction phase. These models are EfficientNetb0 [8], Resnet50 [9], ConvNeXt [10], DenseNet121 [11] and MobileNetV3 [12]. These models are not trained and are used solely to extract features from the dataset. In this way, the cost of the training process is minimized. Feature extraction from deep learning architectures is performed for both lung images and ECG images. Statistical features are calculated for other patient clinical data. In this way, much data from the same patient was used, thus increasing the model’s performance. The feature vector created for each patient is classified using the k-nearest neighbor (KNN) [13], cubic support vector machine (SVM) [14], Logistic Regression (LR) [15], Linear Discriminant Analysis (LDA) [16], Naive Bayes (NB) [17], Random Forest (RF) [18], and Gradient Boosting (GB) [19] classification algorithms. The obtained performance levels were evaluated using different metrics. The most successful model and classifier constitute the proposed model. The proposed model was selected from 5 different pre-trained models and seven different classifiers.

## 3. Experimental Results

In this study, data from a total of 555 patients are used, and three different data types for the same patient and ASA classes are estimated in the four-class dataset. Firstly, the pre-trained models used for feature extraction are EfficientNetB0, Resnet50, ConvNeXt, DenseNet121, and MobileNetV3. In addition to the extracted features, mean, variance, standard deviation, skewness, and kurtosis values are calculated from the patients’ clinical data. As a result of these calculations, a feature vector is created by performing a concatenation operation, and the result is obtained with the classification algorithms. In the experimental studies, chest X-ray and ECG images were resized to 224 × 224 pixels, center cropping was applied, and the images were normalized according to ImageNet pre-training weights. In each pre-trained CNN model, deep features obtained after global average pooling and before the final classification layer were used. The sizes of the resulting feature vectors varied depending on the CNN architecture used, and multimodal feature vectors were created by combining image and clinical features. Features were standardized with StandardScaler, missing values were filled with zeros, and the data was stratified into 80% training and 20% test sets. One hundred estimators were used in the Gradient Boosting classifier, and a random seed of 42 was set for the reproducibility of the experiments. The experiments were performed in Python using the PyTorch/torchvision and scikit-learn libraries, and the version numbers of the libraries were not recorded separately in the code. The results obtained from the experimental studies are presented in Table 1.

When Table 1 is examined, it is evident that GB is the most successful classifier. To more comprehensively evaluate the ordered structure of ASA-PS classes and the classification fit of the models, Matthews Correlation Coefficient (MCC), Cohen’s Kappa, and Quadratic Weighted Kappa (QWK) metrics were also calculated for the Gradient Boosting classifier. While MCC and Cohen’s Kappa evaluate the overall classification fit, QWK considers the ordered relationship between ASA-PS classes and evaluates the agreement of the predictions with the actual classes. The results obtained are presented in Table 2.

As shown in Table 2, EfficientNetB0 achieved the highest values in terms of MCC (0.9634), Cohen’s Kappa (0.9633), and QWK (0.9883). The particularly high QWK value indicates a strong fit between the model predictions and the ordered structure of the ASA-PS classes. The best classifier information and performance values obtained because of the classification of the feature vectors obtained for the pre-trained deep learning models are given in Table 3.

**Figure 4:**
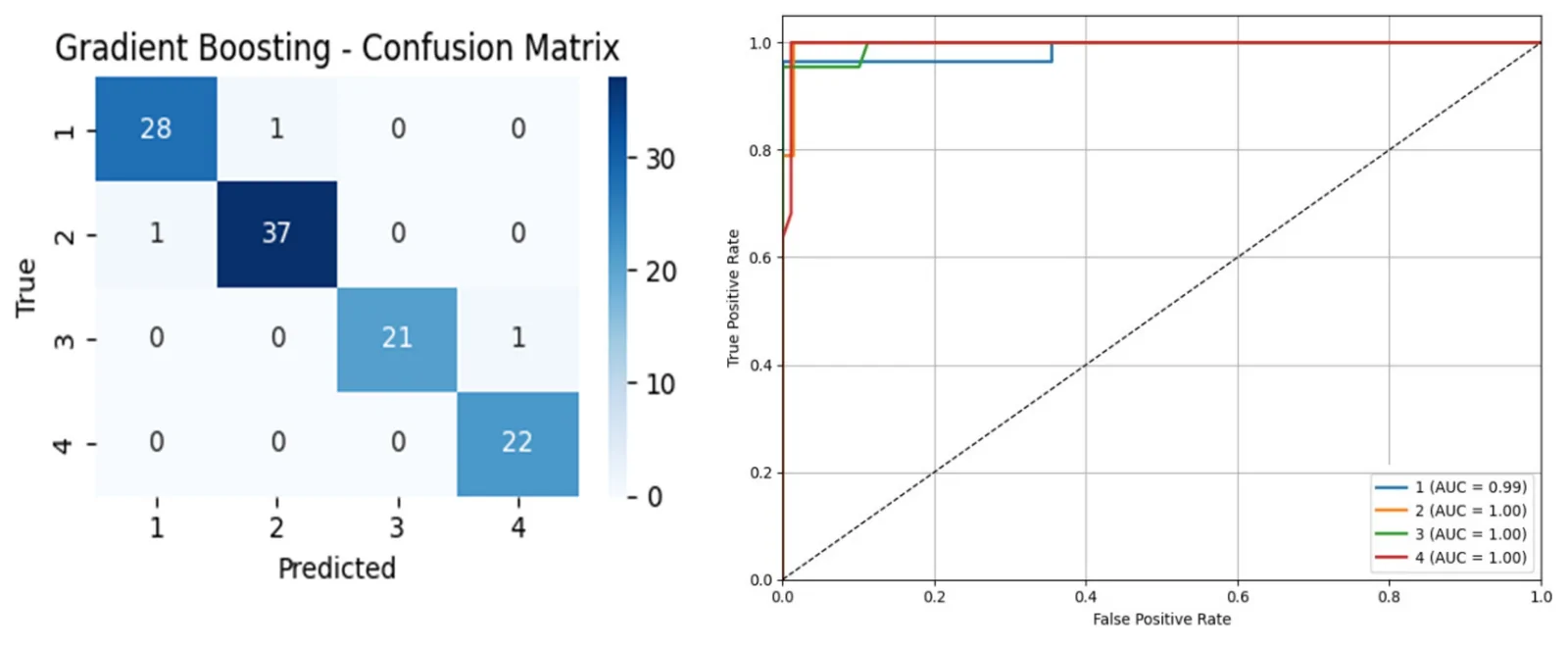
EfficientNetB0 confusion matrix and ROC curve.

As a result of the experimental analysis, high classification success was achieved in four-class ASA prediction using multimodal feature vectors created by combining visual features obtained from digital patient data with different CNN-based pre-trained models, including EfficientNetB0, ResNet50, ConvNeXt, DenseNet121, and MobileNetV3. All models were tested with a Gradient Boosting classifier, and the overall accuracy rates ranged from 95.50% to 97.30%. The EfficientNetB0 model achieved the highest average accuracy of 97.30%. This model demonstrated remarkable success, particularly in the ASA 3 and ASA 4 classes, achieving 100% precision and F1-scores ranging from 97.67% to 97.78%. Although other models generally achieved similar accuracy levels, the DenseNet121 model demonstrated the lowest performance, with an accuracy of 95.50%. However, all models achieved high success in the ASA 3 and ASA 4 classes, indicating that the model can distinguish between serious disease levels more clearly. In the ASA 1 and ASA 2 classes, some models showed minor performance drops. These findings demonstrate that evaluating visual and numerical data together significantly enhances model performance, and the EfficientNetB0 architecture is a highly suitable option for such medical classification tasks. In the continuation of the study, the obtained confusion matrices and ROC curves will be presented for detailed analysis of the classification results, and the discrimination power and general stability of the models will be evaluated in more detail. The EfficinetNetB0 confusion matrix and ROC curve are presented in Figure 4.

**Figure 5:**
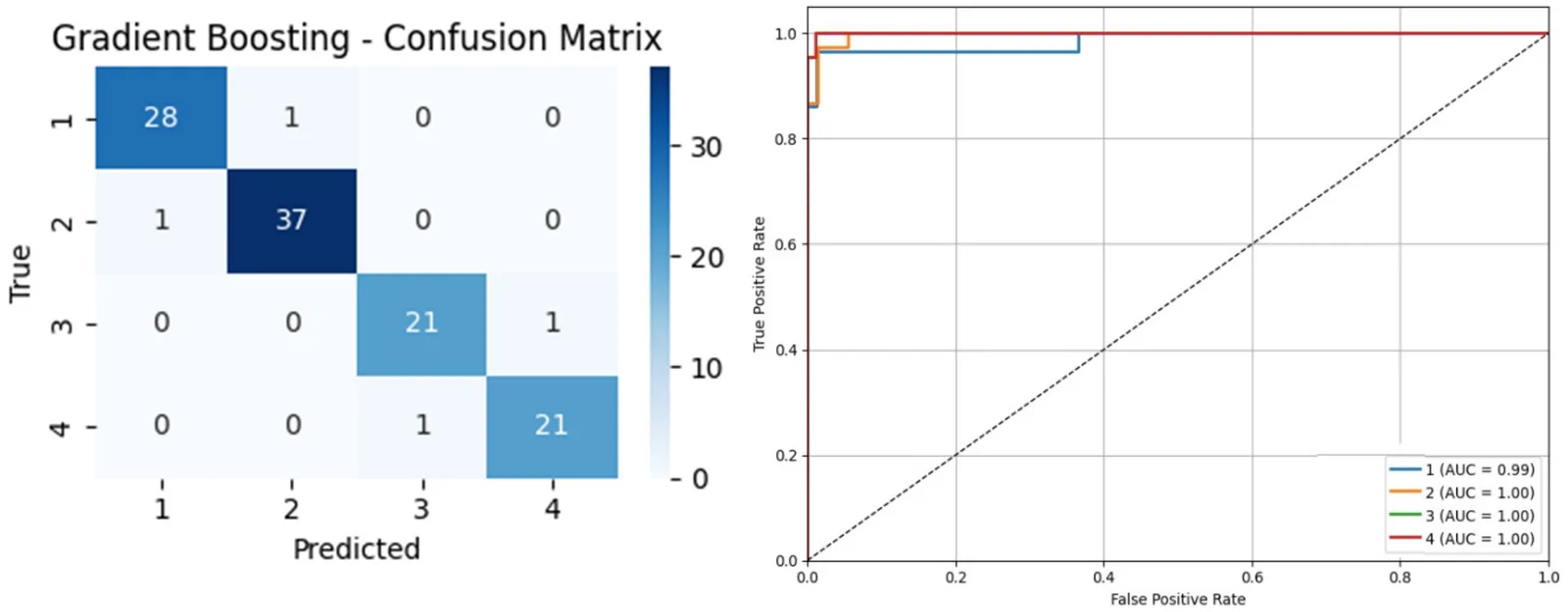
ResNet50 confusion matrix and ROC curve.

When the confusion matrix of the EfficientNetB0 model in Figure 4 is examined, it is seen that the model can distinguish all four ASA classes with very high accuracy. While 28 samples were correctly classified in the ASA 1 class, only 1 sample was confused with ASA 2. All 37 samples were predicted correctly for the ASA 2 class. In the ASA 3 class, 21 samples were correctly classified, and only 1 sample was incorrectly assigned to the ASA 4 class. Similarly, all 22 samples were correctly classified in the ASA 4 class. This distribution indicates that the model exhibits near-perfect discrimination performance, particularly in the ASA 2 and ASA 4 classes, and commits very few errors in the ASA 1 and ASA 3 classes. The fact that the crosstalk between classes is relatively low shows that the model can effectively learn the distinction between the classes. The ROC curves of the model also support this observation. The AUC value for the ASA 2, ASA 3, and ASA 4 classes was calculated as 1.000, indicating that the model exhibits excellent discrimination power in these classes. The AUC value for the ASA 1 class is 0.999, which is also a very high and successful result. The fact that all ROC curves are close to the upper left corner shows that the false positive rates are low and the true positive rates are high. In this direction, the EfficientNetB0 model exhibited strong classification performance with high discrimination power between classes. The ResNet50 confusion matrix and ROC curve are presented in Figure 5.

**Figure 6:**
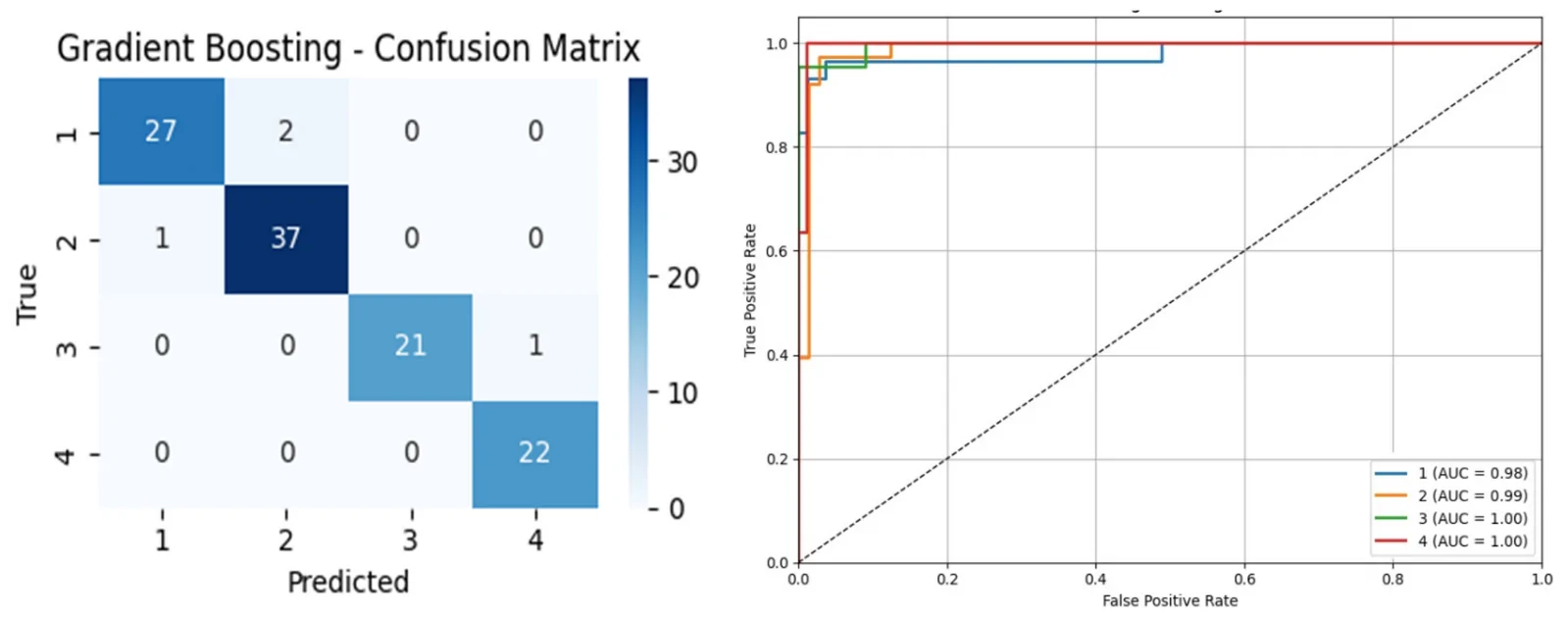
ConvNeXt confusion matrix and ROC curve.

The confusion matrix of the ResNet50 model, presented in Figure 5, shows that the model distinguishes between the four ASA classes with high accuracy. Only one error was made in the ASA 1 and ASA 2 classes, while limited crosstalk was observed between the ASA 3 and ASA 4 classes. ROC curves were calculated as AUC = 1.000 for ASA 2, 3, and 4, and AUC = 0.991 for ASA 1, confirming the high classification success of the model. In general, the ResNet50 model exhibited strong discrimination power and balanced classification performance. The ConvNeXt confusion matrix and ROC curve are presented in Figure 6.

**Figure 7:**
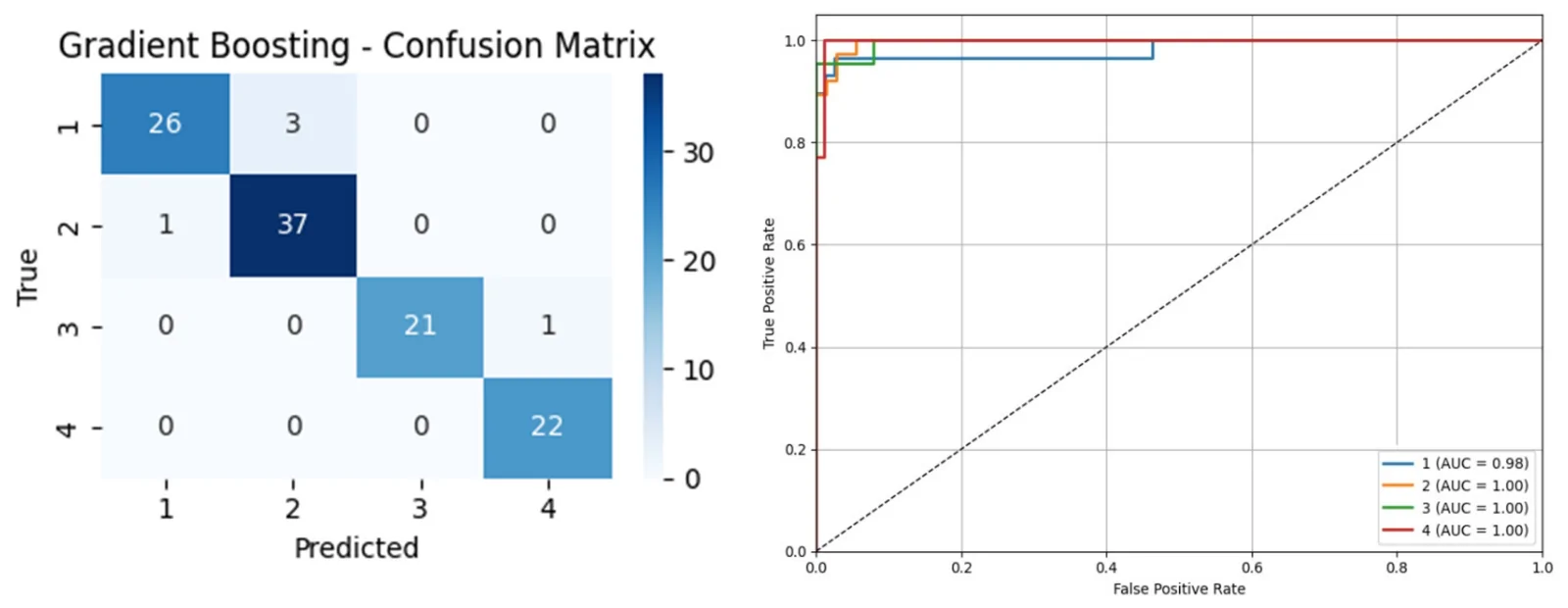
DenseNet121 confusion matrix and ROC curve.

The confusion matrix in Figure 6 indicates that the ConvNeXt model can accurately classify ASA classes 2, 3, and 4 in particular. It was observed that only two examples were assigned to different classes in the ASA 1 class. This shows that the overall accuracy of the model is quite high, but there is limited confusion in the ASA 1 class. When the ROC curves are examined, it is seen that the AUC scores for ASA 2, 3, and 4 are at the ideal level with 1.000, while a high value of 0.988 is obtained for ASA 1. These results show that the ConvNeXt model can reliably distinguish all four classes. The DenseNet121 confusion matrix and ROC curve are presented in Figure 7.

**Figure 8:**
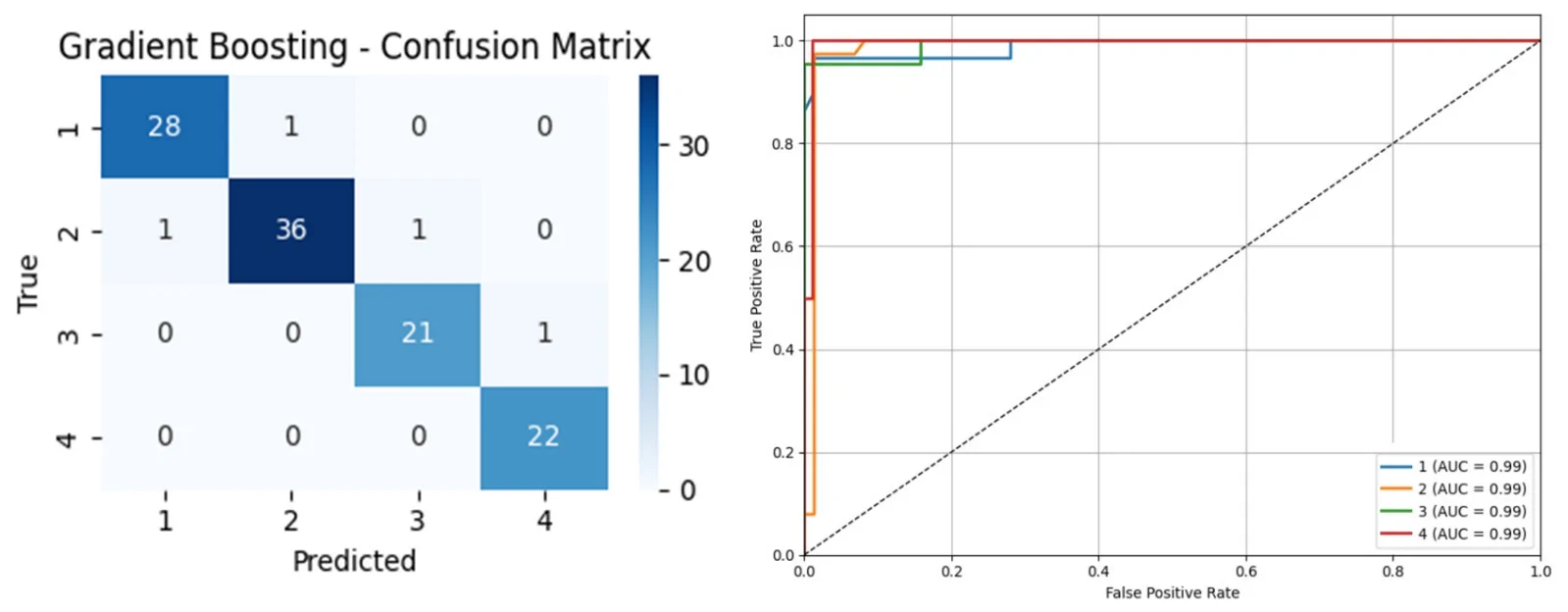
MobileNetV3 confusion matrix and ROC curve.

The confusion matrix in Figure 7 indicates that the DenseNet121 model achieves high accuracy in ASA classes 2, 3, and 4; however, it misclassifies three examples in ASA class 1. The confusion between ASA 1 and ASA 2 is particularly striking. Despite this, the model performs quite well in the ASA 3 and ASA 4 classes, making only one small error each. In the ROC curve analysis, the AUC values of 1.000 for ASA 2, 3, and 4 show that the model provides excellent discrimination in these classes. Although the AUC score for the ASA 1 class is relatively low, with 0.981, it still exhibits strong classification performance. The MobileNetV3 confusion matrix and ROC curve are presented in Figure 8.

The confusion matrix shown in Figure 8 indicates that the MobileNetV3 model accurately classified all examples in the ASA 1 and ASA 4 classes, with very close to the correct classification. In the ASA 2 class, one example was confused with ASA 1, and in the ASA 3 class, one example was confused with ASA 4. Despite these minor deviations, the model’s overall classification success is relatively high. In the ROC curve examination, the AUC values for all classes were calculated as 0.991, indicating that MobileNetV3 has a strong discrimination ability among the four classes. The model demonstrated effective performance due to its lightweight architecture.

As a result of the analyses, it was seen that all deep learning-based models used could successfully distinguish ASA classes. While the EfficientNetB0 model stood out in terms of general accuracy and discrimination power, other models also exhibited strong performance with similarly high AUC values and low classification errors. Particularly in the ASA 2, 3, and 4 classes, significant success was achieved in all models; it was observed that intra-class confusion was generally limited between ASA 1 and ASA 2. The ROC curves confirmed that the models had high discrimination ability among the four classes, and AUC values were at the level of 1.000 for most classes. In conclusion, this multimodal approach, which combines visual and numerical data, demonstrated strong potential for clinical decision support systems by providing high accuracy and reliability in ASA classification.

## 4. Discussion

The determination of ASA-PS, which is used to evaluate the preoperative physical condition and anesthesia risk of patients, can provide consistent and objective results in practice with AI applications that perform analysis in a short time. However, achieving ideal results directly depends on the quality and quantity of data on which the AI model is trained. Incomplete, incorrect, or controversial data may lead the model to make incorrect predictions. All of these may lead to undesirable outcomes, such as inadequate determination of potential risk factors, inappropriate anesthesia techniques, increased risk of complications, and postponement or cancelation of surgery, which may result in increased undesirable mortality and morbidity [20].

Our study presents a novel methodology utilizing AI and ML methods to enhance the objectivity and accuracy of the ASA-PS classification. This approach enabled us to evaluate the risk profile of patients from a multidimensional perspective by integrating not only traditional clinical data but also various imaging data, such as PA Ac and ECG. In particular, the integration of various datasets, including patients’ PA Chest X-ray images, ECG images, biochemical laboratory parameters, examination findings, and anamnesis results, added a scientifically original dimension to our research by analyzing the risk as a whole and made it methodologically sound. To the best of our knowledge, no similar studies in the literature discussed in this section have collated the data in this way and applied the ASA-PS classification.

The ASA-PS classification is utilized in numerous clinical decisions, ranging from surgical planning to prognostication. Xue et al. [21] compared the performance of models using both preoperative and intraoperative data (n = 111,888), independently and combined, in a retrospective cohort study aimed at developing and evaluating ML models to determine the risks of postoperative complications. They also used model-agnostic interpretability methods to provide clinically meaningful interpretations of the models. The best-performing models were obtained by combining preoperative and postoperative data. The homogeneity of the data distribution in the cohort group is also essential. The cohort presented by Lew et al. [22] included oncological patients with a significantly larger proportion of cases with ASA > 3 (i.e., 20% vs. 8%) and a smaller proportion of cases with ASA < 3 (i.e., 13.6% vs. 48.4%). When compared to this study, our model was developed for general surgery populations in ASA 1 (n = 145), ASA 2 (n = 190), ASA 3 (n = 110), and ASA 4 (n = 110). In addition, patient evaluation was performed by the same specialist using the most up-to-date ASA-PS classification to eliminate the differences between anesthesia specialist evaluators.

There are studies on this subject in the literature. Most of these studies used biochemical and demographic data. Wongtangman et al. utilized 35 clinical variables to predict the ASA Physical Status (ASA-PS) classification of patients before surgery. In the study, using clinical data alone resulted in screening a very high number of patients. In this study, the researchers used machine learning methods for ASA prediction. The researchers stated that they were able to predict clinical outcomes, such as 30-day mortality, successfully, as well as the need for intensive care. They also stated that they correctly predicted patients with higher risk than indicated by the anesthesiologist’s assessment in some patient groups [23]. Yoon et al. developed natural language processing (NLP) models to automate ASA-PS classification from pre-anesthesia evaluation notes. They stated that ClinicalBigBird achieved higher accuracy than experts in the field among the models trained with data obtained from 717,389 surgical cases. The AUC value obtained in the study was 91.5%. The researchers stated that the proposed method improved the clinical workflow by providing objective and consistent ASA classification [24]. Lew et al. proposed a machine learning model that automates ASA classification to reduce variability due to individual biases among anesthesiologists. The Logistic Regression model trained with data from 12,064 oncology patients achieved an accuracy value of around 70%. Clinical data were used in this study [22]. Berrigan et al. developed a model that combines ASA PS class with surgeons’ predicted risk estimates to predict the risk of post-surgical complications. An AUC value of 84% was achieved in the study. The researchers stated that the results obtained, clinical decisions, and ASA classification can be powerful tools in postoperative risk assessment [25]. In a study by Birlik et al., a machine learning-based model was developed to predict the risk of postoperative mortality. The developed model provided higher accuracy than traditional models. The researchers presented a more personalized risk classification by highlighting parameters such as creatinine, age, and ejection fraction [26].

A review of the literature reveals the existence of studies conducted using ECG [27], CT [28], and clinical data [29] separately. In the present study, however, ECG, CT, and clinical data were utilized in combination for ASA scoring. It appears that integrating clinical risk classification systems such as ASA scores into machine learning models can enhance predictive power. While previous studies have predominantly relied on clinical data, this study evaluated lung images and ECG data alongside clinical information. Retraining and testing ASA classification via deep learning using features derived from diverse data types (images, signals, and clinical data) has made a significant contribution to the literature in this field.

The generally accepted idea is that AI and ML algorithms have the potential to minimize human-related errors rather than replace the anesthesiologist. Due to their superior abilities in processing large datasets, interpreting images, and analyzing complex patterns, ML algorithms can shorten the clinical decision-making process and optimize resource utilization. They can reduce human labor loss and reduce hospital costs. When evaluated in this respect, it becomes apparent that they offer a multifaceted benefit. The use of ML to investigate large datasets in anesthesia seems promising in morbidity and mortality risk prediction [30]. Zhang et al. aimed to improve this situation by developing an automated system that predicts ASA-PS with higher precision based on patients’ comorbidities and medications. They ultimately demonstrated that the model used in this study can predict ASA-PS with an accuracy similar to that of anesthesiologists [31].

## 5. Limitations

The retrospective and single-center nature of the study, leading to deficiencies in data quality and limitations in the generalizability of the model; obtaining ECG images from physical files and photographing them, making data standardization difficult; the ASA-PS labels on which the model was trained containing human subjectivity; and data imbalance between classes are limitations that may affect the accuracy of the model. Future studies should include external validation using independent datasets from different institutions and patient populations.

## 6. Conclusions

In this study, a deep learning-based classification model utilizing multimodal data was developed to reduce the dependence of the American Society of Anesthesiologists Physical Status Classification (ASA-PS) system on subjective evaluations and to provide a more consistent structure in clinical decision-making processes. The use of various types of data, including lung images, ECG data, and clinical parameters, provided a more comprehensive and holistic assessment of patients. In this way, high success was achieved in predicting ASA class. In particular, the combined analysis of the features obtained from these three types of data significantly enhanced the predictive power of the model, yielding a high accuracy value of 97.30%. This obtained accuracy value will allow the developed system to determine patients’ risk before surgery more objectively.

Additionally, it will enable the development of patient-specific treatment plans. In addition, the model’s successful results in advanced metrics, such as the ROC curve, precision, and F1 score, demonstrate that the system not only exhibits general accuracy but also strong performance in class discrimination. As a result, the study will support traditional clinical evaluations and will reduce the margin of error and uncertainty. Future work will focus on strengthening the proposed model by utilizing larger and more diverse datasets obtained from various clinical centers. Furthermore, analyses incorporating data from multiple institutions and expert evaluations will be conducted to examine the model’s generalizability and reliability in greater depth.

## Figures and Tables

**Table 1:** Accuracy values of models in different classifiers for ASA score prediction (%).

Models	KNN	SVM	LR	LDA	NB	RF	GB
EfficientNetB0	54.05	42.34	68.47	81.08	61.26	76.58	97.30
ResNet50	42.34	35.14	64.86	68.47	38.74	80.18	96.40
ConvNeXt	54.95	49.55	63.96	86.49	64.86	85.59	96.40
DenseNet121	51.35	45.95	65.77	84.68	57.66	83.78	95.55
MobileNetV3	45.95	42.34	66.67	85.59	61.26	84.68	96.40

**Table 2:** Agreement and ordinal performance metrics of pretrained models using the Gradient Boosting classifier.

Model	MCC	Cohen’s Kappa	QWK
EfficientNetB0	0.9634	0.9633	0.9883
ResNet50	0.9510	0.9510	0.9842
ConvNeXt	0.9512	0.9510	0.9843
DenseNet121	0.9392	0.9387	0.9802
MobileNetV3	0.9512	0.9511	0.9844

**Table 3:** Performance comparisons of models in ASA score prediction (%).

Models	Classifier	Average Accuracy	Class	Precision	Recall	F1-Score
EfficientNetB0	GB	**97.30**	ASA 1	96.55	96.55	96.55
			ASA 2	97.37	97.37	97.37
			ASA 3	100	95.45	97.67
			ASA 4	95.65	100	97.78
Resnet50	GB	96.40	ASA 1	96.55	96.55	96.55
			ASA 2	97.37	97.37	97.37
			ASA 3	95.45	95.45	95.45
			ASA 4	95.45	95.45	95.45
ConvNeXt	GB	96.40	ASA 1	96.43	93.10	94.74
			ASA 2	94.87	97.37	96.10
			ASA 3	100	95.45	97.67
			ASA 4	95.65	100	97.78
DenseNet121	GB	95.50	ASA 1	96.30	89.66	92.86
			ASA 2	92.50	97.37	94.87
			ASA 3	100	95.45	97.67
			ASA 4	95.65	100	97.78
Mobilenetv3	GB	96.40	ASA 1	96.55	96.55	96.55
			ASA 2	97.30	94.74	96.00
			ASA 3	95.45	95.45	95.45
			ASA 4	95.65	100	97.78

## Data Availability

The data used in this research are available upon request from the corresponding author.
